# Design and Evaluation of a new mechatronic platform for assessment and prevention of fall risks

**DOI:** 10.1186/1743-0003-9-51

**Published:** 2012-07-28

**Authors:** Lorenzo Bassi Luciani, Vincenzo Genovese, Vito Monaco, Luca Odetti, Emanuele Cattin, Silvestro Micera

**Affiliations:** 1 The BioRobotics Institute, Scuola Superiore Sant’Anna, P.za Martiri della Libertà, 33 – 56127, Pisa, Italy; 2 Tecnalia, Pisa, Italy; 3 Translational Neural Engineering Laboratory, Center for Neuroprosthetics and Institute of Bioengineering, School of Engineering, Swiss Federal Institute of Technology Lausanne (EPFL), Lausanne, Switzerland

**Keywords:** Mechatronics, Locomotion perturbation, Falling, Calibration, Balance control, Event triggering

## Abstract

**Background:**

Studying the responses in human behaviour to external perturbations during daily motor tasks is of key importance for understanding mechanisms of balance control and for investigating the functional response of targeted subjects. Experimental platforms as far developed entail a low number of perturbations and, only in few cases, have been designed to measure variables used at run time to trigger events during a certain motor task.

**Methods:**

This work introduces a new mechatronic device, named SENLY, that provides balance perturbations while subjects carry out daily motor tasks (e.g., walking, upright stance). SENLY mainly consists of two independently-controlled treadmills that destabilize balance by suddenly perturbing belts movements in the horizontal plane. It is also provided with force sensors, which can be used at run time to estimate the ground reaction forces and identify events along the gait cycle in order to trigger the platform perturbation. The paper also describes the customized procedures adopted to calibrate the platform and the first testing trials aimed at evaluating its performance.

**Results:**

SENLY allows to measure both vertical ground reaction forces and their related location more precisely and more accurately than other platforms of the same size. Moreover, the platform kinematic and kinetic performance meets all required specifications, with a negligible influence of the instrumental noise.

**Conclusion:**

A new perturbing platform able to reproduce different slipping paradigms while measuring GRFs at run time in order to enable the asynchronous triggering during the gait cycle was designed and developed. Calibration procedures and pilot tests show that SENLY allows to suitably estimate dynamical features of the load and to standardize experimental sessions, improving the efficacy of functional analysis.

## Background

Fall prevention is currently a very important, social, and economical problem due to the aging of the population worldwide. Given the strong relationship between health and fall risk, a large number of exercise programs, aimed at enhancing strength, endurance and body mechanics of targeted subjects, have been proposed to avoid the traumatic consequences of such occurrence. The final goal of these treatments is to significantly reduce fall accidents and related effects in order to improve the independence of individuals, and reduce social costs due to hospitalization [1,2].

A wide range of devices that simulate different kinds of falls have been developed to investigate human behavior during the perturbation of balance control. These devices can be classified into three main groups: *i.* platforms aimed at perturbing the quiet upright stance by means of tilts, translations and rotations of the support base; *ii.* treadmills that destabilize subjects while walking; *iii.* complex systems generating unexpected perturbations due to slipping surfaces or suddenly appearing obstacles, which occur while subjects are carrying out daily motor tasks such as walking or sit-to-stand.

The analysis of postural perturbations while subjects keep an upright stance has been developed to understand the effects of age-related impairments and training on the balance control system [3,4], to study compensatory arm responses to externally applied postural destabilization [5], and to provide clinical assessments or therapeutic training for subjects affected by lack of balance control [6]. Studies concerning perturbed treadmill locomotion have been carried out to analyze trained compensatory postural responses in older adults [7,8], to investigate stumbling reaction in young and elderly people [9,10], and to describe limb coordination of healthy subjects while locomotion is perturbed by the movement of one belt [11]. Other authors have also developed special devices embedded into the walkway in order to define standardized stability tests, to explore the recovery of gait stability in healthy individuals after a slipping perturbation [12], or to analyze the role of the arms while keeping balance after the sudden appearance of an obstacle [13]. Finally, concerning unperturbed motor tasks, previous studies provide insights concerning how to accurately detect features related to the interaction between feet and instrumented treadmill [14,15].

All devices described in literature allow researchers to analyze the biomechanics of falls in a standardized framework, while kinematics, kinetics, and muscle activation are recorded. The aim of this paper is to introduce a new mechatronic platform, named SENLY (“SENsorized perturbing platform for fall assessment and prevention for eLderly and Young subjects”), which is able to provide balance perturbations while subjects carry out daily motor tasks (e.g., quiet upright stance, walking). SENLY destabilizes balance control by using a pair of independently-controlled belts which can move in the both anterior-posterior (AP) and medial-lateral (ML) directions in order to perturb one or both feet. It is provided with force sensors that allow to measure vertical Ground Reaction Force (GRF) components acting under each foot, and to estimate the Center Of Pressure (COP) related to each GRF. Noticeably, GRFs are processed at run time in order to trigger events along the gait cycle.

Compared to other platforms [5-11], the main innovative feature of SENLY is the integration of its ability to provide mono and bi-lateral perturbations of the support base in all horizontal directions during a wide range of motor tasks, with the possibility to record external forces that act on subjects and that can be used to standardize experimental protocols. In particular, SENLY accounts for technical solutions which allow to implement almost all experimental protocols as far carried out by using different platforms (e.g., perturbing balance control while keeping the upright stance or walking, providing disturbance on one or both feet, delivering perturbations toward both AP and ML directions, triggering different phases of the gait cycle). As a result, SENLY appears to be one of the most versatile platforms yet developed for analyzing perturbed balance control during dynamic motor tasks. The paper reports choices leading to the platform design, and describes mechatronic structure, calibration procedures, and performance.

## Design and development of SENLY

### Design criteria

In literature [16] three main accidental causes of falling are identified: slipping, tripping, and missteps. Among these, slipping and tripping involve the greater amount of accidents [17,18]. With regard to slipping, a hazard exists when the coefficient of friction between the contact surfaces is inadequate to sustain the reasonably expected ambulation dynamics. During walking, a slip usually occurs at or immediately after the heel-strike, involving a sudden movement of the slipping foot toward multiple directions [16,19]. This occurrence has been shown to account for approximately 62 % of underfoot accidents [20], and requires a great amount of ankle torque [21] which is strongly impaired due to natural ageing [22]. Therefore, a platform aimed at reproducing horizontal perturbations of one or both feet within a standardized framework allows to investigate how the balance control system manages one of the most frequent accidental perturbations.

For these reasons, SENLY was designed to provide perturbations of the balance control system via belts slipping in horizontal directions (i.e., right foot 0°-180°, left foot 180°-360° relative to the plane of progression) while subjects carry out daily motor tasks. Belts had to be independently controlled along AP and ML directions and the platform had to be designed to allow measurement of the vertical component of the force of interaction between subjects and floor.

In order to define the main kinematic specifications, we used previously published data. In particular, McIlroy and Maki [23] applied perturbations characterized by maximal acceleration of 3 m/sec^2^, maximal velocity of 0.9 m/sec and maximum displacement of 270 mm, which represent a suitable compromise among other approaches [3,8,12]. Therefore, SENLY was designed to guarantee a perturbation of 300 mm in less than 0.5 sec.

### Mechatronic platform

**Figure 1:**
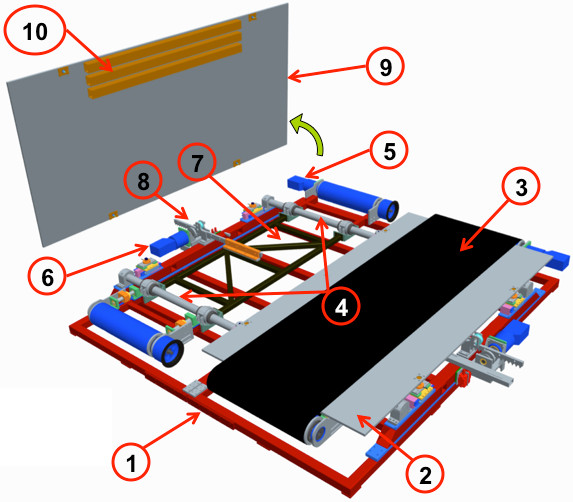
**Exploded view of SENLY.** The figure shows: the support base of the main frame (**1**), the right sensorized surface (**2**), the right belt (**3**), the two cylindrical rails (**4**), the AP (**5**) and ML (**6**) actuation systems of the left treadmill, the mobile frame (**7**) and the pinion-rack system related to the left side (**8**); the tilted left sensorized surface (**9**); the stiffening rectangular bars (**10**). Homologous components belonging to the contralateral sides and the security frame are not shown to make reading easier. See Figure 6 for more details.

SENLY is basically composed of two symmetrical sectors, each consisting of a treadmill which belt wraps a sensorized flat surface (Figure 1). Each treadmill is a mobile frame provided with two rollers and a pre-tensioned belt in which the tension can be regulated by a belt tensioning system. The mobile frame rigidly links the distance between roller axes and provides mounting for brushless motors (JVL MAC800 750 W) driving the AP motion of the belt. Two cylindrical rails are connected to the main frame and pass through four linear bearings for each treadmill frame, allowing them to independently translate in the ML direction. Given this technical solution, belts are spaced from each other at a distance of less than 10 mm, enabling a more comfortable gait than other solutions [24].

**Figure 2:**
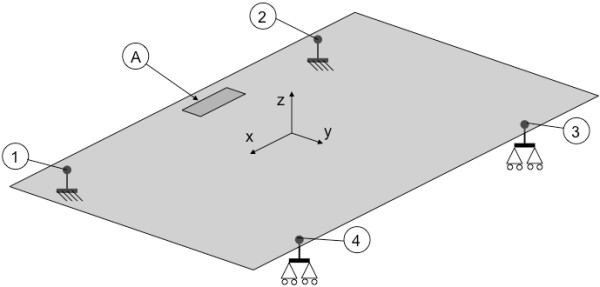
**Schematic representation of sensorized surface related constraints.** The figure shows the location of load cells on the right sensorized surface. The three-axial load cells (**1**, **2**) are fixed to the ground (cylindrical rails) and each of the mono axial cells (**3**, **4**) is connected to the frame by a two degrees of freedom free rail system. All tips of the load cells are connected to the sensorized surface by spherical joints. Areas A represent the rectangular extent where the load was applied during the FEA. In particular, A represents the area used by a subject while walking across both treadmills.

A flat sensorized surface, suitably shaped and made in aluminum (Figure 1), is located between each belt and related treadmill frame. Each flat surface is connected to the main frame by four load cells via spherical joints: two three-axial load cells (Michigan three-axial load cell Mod. TRD-3A) are fixed on the cylindrical rails, and two mono axial cells (Metior mono-axial Mod. CVK 5KN) are connected to the main frame by a free rail system (Figure 1). Figure 2 shows the schematic representation of the mechanical model of the sensorized surface. Each load cell has been calibrated and certified by the supplier*.* Data from the load cells are acquired by the Vishay Micro-Measurements System 7000 Data Acquisition System, with a sample rate of 1000 Hz.

The load cell set (Figure 2) related to each sensorized surface allows to measure the three orthogonal components of applied force and moment. The COP is estimated in accordance with previous literature [25,26] by means of the equilibrium of moments of the surface, assuming that the point of application of the force between foot and belt (thickness 2 mm) is vertically projected to the sensorized surface.

**Figure 3:**
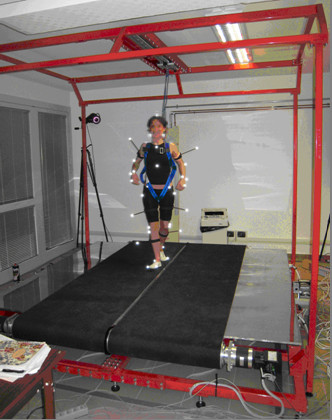
**Picture of a subject while being on SENLY.** SENLY with a tester attached to the tubular safety structure.

The ML motion of each treadmill is actuated by a brushless motor (JVL MAC800 750 W) and carried out by a pinion-rack system, where the rack is connected to the treadmill frame and the pinion to the main frame. Each treadmill is provided with two limit switches (Honewell GLS series), one for the zero position and one for emergency stop. All parts constituting the frames are made in steel. SENLY is also provided with a tubular safety structure to which the tester, wearing a harness, is attached by means of a damper-cable-snap-hook system (Figure 3).Technical features of the entire platform are summarized in Table 1.

**Table 1:** Technical features of SENLY

Length	2500 mm
Width	2500 mm
Overall height (with tester security frame)	3000 mm
Walkway height	300 mm
Max tester weight	110 kg
Max tester height	1900 mm
Treadmill belt width	750 mm
Treadmill weight	110 kg
Sensorized surface dimension	2290x1250x15 mm
AP direction: max velocity	1.8 m/s
AP direction: max acceleration	8 m/s^2^
ML direction: max displacement	300 mm
ML direction: max velocity	1.25 m/s
ML direction: max acceleration	2.4 m/s^2^

The table accounts for sizes of the platform, maximum load allowed to carry out exercises on SENLY, and maximum performance, in term of kinematics, of belt movements.

### Design of the sensorized surface

Sensorized surfaces were shaped and dimensioned after defining maximum acceptable deformation and frequency response. In particular, the goal of the Finite Element Analysis (FEA) was to keep both the deformation due to the maximum load (3000 N) below 1 mm, and the first mode of vibration above 20 Hz [27]. FEA was carried out using ANSYS TM software.

The 3D model implemented in the FEA used shell elements. Nodes corresponding to three (see 1 and 2 in Figure 2) and mono (see 3 and 4 in Figure 2) axial load cells were constrained against respectively the translation toward x, y, and z directions, and the translation toward the z direction. The vertical load was applied over a rectangular extent (160x60 mm) representing the interface between contact foot and sensorized surface (see foot contact area in Figure 2). This load was applied on the walking area (i.e., the extent of the surface used by a subject while walking across both treadmills; see Figure 2).

According to these specifications, each surface consisted of a sheet of aluminum (2290x1250x15 mm) provided with three rectangular bars (50x60x1500 mm, 4 mm thick) which were asymmetrically glued (Plexus MA832 series) under the walking area (Figure 1). The total mass of each sensorized surface is 120 kg. This configuration allowed to keep the deflection as low as 0.87 mm, with a first mode of vibration at 20.54 Hz.

### Motion Control

**Figure 4:**
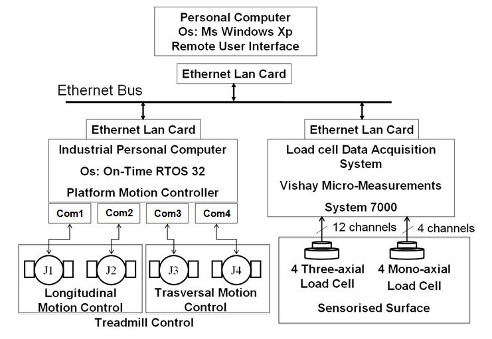
Motion Control Diagram.

The control of SENLY (Figure 4) is distributed over two nodes connected to an Ethernet bus. The first node, a Personal Computer (Pc) running under Microsoft Windows Xp, hosts the Remote User Interface. The second node, an industrial PC, running under the real-time operating system On Time RTOS-32, hosts the Platform Motion Controller. Force sensors are managed by a third node, represented by the Vishay Micro-Measurements System 7000.

Communications between the Remote User Interface, Platform Motion Controller, and data acquisition system are based on Ethernet UDP. The industrial PC is connected via four RS232 serial-links to the four integrated servomotors guiding both the AP motion of the belts and the ML motion of treadmill frames.

Each servomotor controls the speed of each joint as specified by commands coming from the Platform Motion Controller.

### The remote user interface

The Remote User Interface is a graphical application, designed and developed for an expert user, who is in charge of the setup of all the phases and operations of an end user session with the platform. This is done by suitably adjusting a set o parameters referring to:

speed and position profiles of all treadmill degrees of freedom;

real-time pre-processing of measured forces;

perturbation triggers;

calibration procedure.

Pre-processing of measured forces is performed by the Motion Controller before computing GRFs and COPs, and consists of:

canceling the offset related to the load of force plates;

applying a digital filter based on the cascade of a low-pass Butterworth and a band-stop notch filters to reduce the contribution of noise;

applying a calibrating matrix to increase the accuracy of the measure.

The offsets can be computed by averaging records on each acquisition channel along a time window lasting between 1 ms and 5 s. The operator can set both the order and the cut-off frequency of the low-pass Butterworth filter, as well as its related Q factor, and the center frequency of the notch filter.

Trajectory implementation is based on the decomposition of the speed/position profiles in sequences (one for each joint) of speed/position set points and their related timing.

Finally, the Remote User Interface allows the status of the platform to be visualized and/or saved at run-time, in terms of speed, position, and torque for each joint, GRFs and COPs. The Remote User interface was developed using Microsoft Visual basic 6.0.

The Platform Motion Controller receives features related to desired kinematic profiles, and turns them into a sequence of Speed/Position - Acceleration commands that are sent to servomotors at the right time. Basically, the main purpose of the controller is to schedule commands for the servomotors in accordance with timing. The RTOS-32 supports real-time multi-tasking in a concurrent and cooperative manner leading to highly efficient development of activity scheduling.

Other tasks assigned to the control system are:

pre-processing of measured data forces according to the setup of the Remote User Interface, and estimation of COP;

acquisition of the state of the platform, in terms of treadmill kinematics and related GRFs, and sending them, suitably formatted, to the Remote User Interface;

management of abnormal device behaviors and associated security measures.

The Platform Motion Controller was developed using Microsoft Visual C++ 6.0.

### Perturbation trigger

Perturbations of treadmill kinematics can be activated either synchronously, that is, simultaneously with the Start command run by the user, or asynchronously, that is, when a particular load distribution on both platforms is detected. In particular, according to the distribution of the GRF vertical components between the feet and to their related trends (i.e., rising or falling), initial contact, loading response, mid stance, terminal stance, and pre-swing can be approximately estimated, in accordance with data reported in literature [28]. Triggering can be set up by a set of parameters accounting for: subject weight, perturbed foot, and ratio between GRF vertical component trends. The algorithm compares data at the current instant *t*_*i*_ and at *t*_*i-1*_ and triggers a specific event when a suitable condition set is verified. For instance, the set of conditions that lead to recognizing the early stance of the left foot are those described by the relationships (1):

(1) $$\left\{ \begin{array}{c} {\text{F}}_{\text{slope}}=sign\left({\text{FzLeft}}_{\text{i}}-{\text{FzLeft}}_{\text{i}-1}\right)\\ \frac{{\text{|FzLeft}}_{\text{i}}\text{|}}{{\text{|FzRight}}_{\text{i}}|}>\text{Kp-dk}\\ \frac{{\text{|FzLeft}}_{\text{i}}\text{|}}{{\text{|FzRight}}_{\text{i}}|}<\text{Kp}+\text{dk} \end{array}\right.$$

where:

Fslope is the time rate of change (positive or negative) of the force signal;

FzLeft and FzRight are the GRF vertical components under, respectively, the left and the right foot;

Kp is the ratio between the GRF vertical components set by the user;

dk represents a threshold band around Kp.

According to Eq. 1,: the first condition detects whether the left foot is in loading response; second and third conditions detect the instant in which the ratio is within the threshold band.

**Figure 5:**
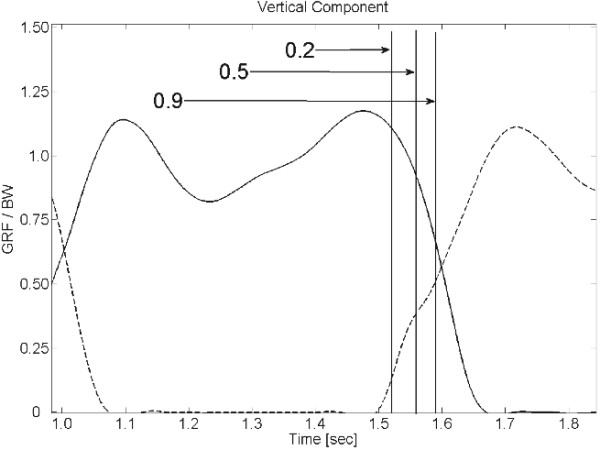
**Triggering.** GRF vertical component related to the right (solid line) and left (dashed line) foot, measured by SENLY during the gait; vertical bars identify the instants in which the ratio between the GRF vertical components of the left and right foot assumes values respectively of 0.2, 0.5, and 0.9 during the loading response of the left foot.

Asynchronous activation is required when the vertical component of both GRFs referring to a specific stance phase is expected to be triggered in order to provide a standard perturbation to all subjects involved in the experimental session. Figure 5 shows a representative example of asynchronous triggering.

## Calibration

### Materials and methods

In order to reduce inaccuracies on the measure of amplitude, direction, and location of the applied force due to the cross-talk among channels, the calibration matrix related to each platform was estimated as described by previous authors [29]. In literature many guidelines leading to the calibration of force plates have been proposed and can be basically classified into static and dynamic approaches. Static calibration consists of loading the platform with masses of known weight located in known positions [30,31]. This approach estimates the spatial accuracy of the measurement across the platform yet it is time consuming, and can be affected by load positioning inaccuracies [27,32]. Dynamic calibration was therefore developed in order to simplify procedures by using intrinsically movable loads and so avoid moving masses manually and related errors [27,32,33]. Another approach, proposed by Collins and colleagues [24], consists of calibrating force plates by using an instrumented pole of known weight, and provided with reflective markers. The instrumented pole measures applied load magnitudes through a single-axis load cell, and load directions through motion capture markers, thus enhancing the accuracy of the calibration matrix.

In our case, all these methods cannot be applied to SENLY because they require motion capture systems [24,33], are based on movable platforms with reduced workspace compared to SENLY [32], and are all based on fixed weight load [24,27,30-32]. This latter issue entails that almost all these calibration approaches do not account for platform accuracy and precision when loaded by dynamic loads, that is, they do not account for the dynamic response of force sensors. Conversely, dynamic approaches reported in literature either adopt quite slow (e.g., 1–2 Hz) movements [24,33] with respect to the platform frequency band, or their weights are light [32], introducing potential inaccuracies [34]. Actually, from the best of our knowledge, only Paolini and colleagues [35] highlighted the importance of evaluating the performance of an instrumented treadmill in the presence of forces typically applied during gait, that is variable forces which power spectral density is characterized by frequencies higher than 1–2 Hz.

**Figure 6:**
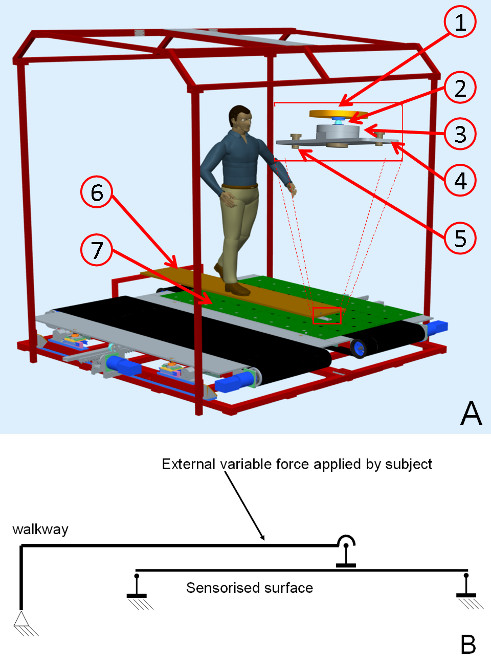
**Calibration procedures.** Top panel: CAD view of calibration procedures: the operator on the walkway (**6**) loads the three-axial load cell (**3**) and consequently the force platform. The referring position plate (**7**), clamped on the sensorized surface, allows to locate the load cell (**3**), with known angular reference by means of pivots (**5**) mounted on the load cell support (**4**); the gripper disc (**1**) is stuck under the walkway and hosts a spherical joint (**2**) connecting the load cell. Bottom panel: schematic representation of the mechanical model during the calibration procedure; noticeable, the load cell measures the same force that the walkway applies to the sensorized surface.

For these reasons, a specific calibration protocol was defined and applied to set up the calibration matrix of each sensorized platform. It mainly consisted of comparing GRF and COP components estimated by SENLY to those related to a three-axial load cell placed between force plates and an external walkway, by means of a removable reference grid (Figure 6). The load cell-walkway interface was a gripped disc fixed to the sensor by a spherical joint to avoid torque transmission. The reference sensor was a Michigan three-axial load cell fixed on a support allowing localization on the grid with respect to the angular reference frame. The reference grid was a 2100x1050 mm sheet of aluminium, which was clamped to the sensorized surface.

During the calibration, a subject was asked to load the walkway while moving for a ten-seconds-long trial. For each of the two plates, the data acquisition procedure accounted for records in k = 9 known positions that span all the surface.

**Figure 7:**
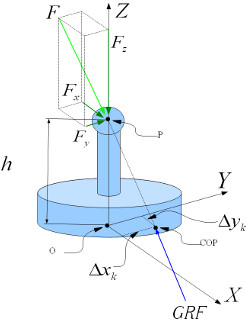
**Free Body diagram of the load cell.** The force *F*, whose components are *Fx*, *Fy* and *Fz*, applied at **P**, is equilibrated by GRF applied at **COP** along the same direction of *F*.

Since the distance between the spherical joint and the sensorized surface was h = 50 mm, the point of application of the force exerted by the sensorized surface to the load cell (see **COP** in Figure 7) does not coincide with the vertical projection of the spherical joint (see **P** in Figure 7) on the ground (see **O** in Figure 7). Therefore, the location of the GRF is given by:

(2) $$\begin{array}{l} COP_{x}^{k}=x_{k}+\Delta x_{k}\\ COP_{y}^{k}=y_{k}+\Delta y_{k} \end{array}$$

where:

COPkx and COPky are the components of the point of GRF related to the kth reference position;

xk and yk are the coordinates of the vertical projection of the spherical joint on the surface (see O in Figure 7) related to the kth reference position;

Δxk and Δyk are the components of the distance between GRF (see COP in Figure 7) and vertical projection of the spherical joint on the sensorized surface (see O in Figure 7), related to the kth reference position.

According to the force equilibrium shown in Figure 7, *Δx*_*k*_ and *Δy*_*k*_ can be estimated by the following Equations:

(3) $$\begin{array}{l} \Delta x_{k}=h\cdot F_{x}/F_{z}\\ \Delta y_{k}=h\cdot F_{y}/F_{z} \end{array}$$

where *F*_*x*_, *F*_*y*_ and *F*_*z*_ are the components of the applied force.

The calibration algorithm was then based on the least-squares approach, and aimed at estimating the 36 parameters constituting the calibration matrix resulting from the model described in (4):

(4) $$\begin{array}{c} \left[\begin{array}{c} {\bar{F}}_{\mathrm{app}}\\ {\bar{M}}_{\mathrm{app}} \end{array}\right]=C\left[\begin{array}{c} {\bar{F}}_{\mathrm{plat}}\\ {\bar{M}}_{\mathrm{plat}} \end{array}\right];\\ \begin{array}{cc} {\bar{F}}_{\mathrm{app}}=\left[\begin{array}{c} Fx_{\mathrm{app}}\\ Fy_{\mathrm{app}}\\ Fz_{\mathrm{app}} \end{array}\right]; & {\bar{M}}_{\mathrm{app}}=\left[\begin{array}{c} Mx_{\mathrm{app}}\\ My_{\mathrm{app}}\\ Mz_{\mathrm{app}} \end{array}\right] \end{array}\\ \begin{array}{cc} {\bar{F}}_{\mathrm{plat}}=\left[\begin{array}{c} Fx_{\mathrm{plat}}\\ Fy_{\mathrm{plat}}\\ Fz_{\mathrm{plat}} \end{array}\right]; & {\bar{M}}_{\mathrm{plat}}=\left[\begin{array}{c} Mx_{\mathrm{plat}}\\ My_{\mathrm{plat}}\\ Mz_{\mathrm{plat}} \end{array}\right] \end{array}; \end{array}\text{;}$$

where:${\bar{F}}_{\mathrm{app}}$

${\bar{F}}_{\mathrm{app}}$F¯app is the applied force measured by the reference sensor;

${\bar{F}}_{\mathrm{app}}$M¯app is the moment generated by F¯app and calculated with respect to the center of the reference frame related to the platform;

${\bar{F}}_{\mathrm{plat}}$F¯plat and M¯plat are respectively estimated force and moment;

${\bar{M}}_{\mathrm{plat}}$C is the 6x6 calibration matrix.

For each *k* position, a 10 s long record was acquired with 1000 Hz sample rate, involving 10x1000x6 non linear relations. The algorithm, therefore, estimated the 36 parameters minimizing the Root Mean Square (RMS) of the residual error.

### Results of calibration

**Figure 8:**
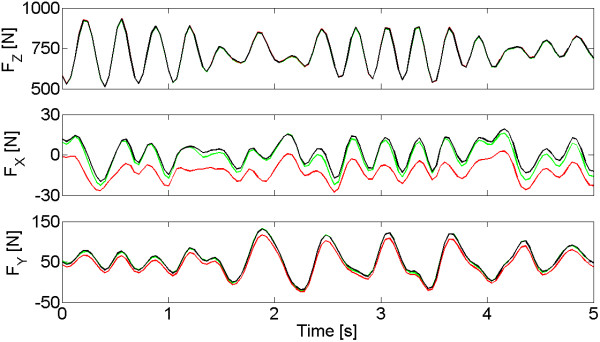
**Recorded GRF after calibration.** Representative 5-seconds-long record related of the set of forces developed by the operator while moving on the walkway during calibration procedures. The figure shows the components of GRF estimated by SENLY before (red) and after (green) calibrating, and compares them to those measured by the reference load cell (black). x, y and z axes are respectively the AP, ML and vertical directions.

The movement of the subject on the walkway during calibration procedures generated variable forces which vertical and horizontal components ranged respectively between 500 N and 1000 N, and −50 N and 150 N (Figure 8). COP also deviated of about 15 mm from the centre of the load cell (see **O** in Figure 7).

As expected, calibration improved both precision and accuracy of the measurement, decreasing RMS and maximum error and increasing correlation coefficients between applied and estimated variables, as reported in Table 2. Moreover, it allowed to achieve a better estimation of measurements than those adopted for other platforms of comparable size [15].

**Table 2:** Comparison between performance before and after calibration

	**RMSE**		**Correlation Coefficient**	
	**Before**	**After**	**Before**	**After**
Fx [N]	8,34	1,98	0,99733	0,99837
Fy [N]	14,34	4,14	0,81483	0,93236
Fz [N]	7,17	4,13	0,99879	0,99955
COPx [mm]	3,11	1,52	0,99992	0,99995
COPy [mm]	3,82	1,68	0,99991	0,99994

The table reports RMSE and Correlation Coefficients between applied and estimated load, both force and point of application.

## SENLY Testing

This section is firstly aimed at describing tests carried out to verify both the consistence of expected performance of the sensorized surface with actual ones, and the influence of both the instrumental noise and the noise due to moving belts. In order to characterize the noise (*n*), its power spectral density, labeled as Gn(f), was calculated using Welch’s method as described by Paolini and colleagues [35]. Moreover, the total power of *n* was estimated by integrating Gn(f) over frequency, up to the value at which 99% of the total power was reached.

Finally the section also describes a set of pilot tests carried out to verify the attitude of the asynchronously trigger to detect a specific event of the gait cycle. All data adopted for this section have been recorded after the calibration procedure with a sample rate of 1000 Hz.

### Analysis of the first mode of vibration of the sensorized surface

In order to verify the occurrence of the first mode of vibration, the natural frequency of each sensorized surface was estimated by observing the impulsive response of the vertical component of the GRF. The impulse was generated by hammering each platform in 50 randomly selected points spread out on each whole surface. The temporal distance between 2 consecutive strokes was at least 2 minutes. Results showed that the first mode of vibration occurred for frequency higher than 20 Hz. Moreover, in most of the cases (about the 95%), it occurred between 20.5 Hz and 20.9 Hz, confirming expected performance obtained by the FEA.

### Instrumental noise

The power spectral density (Gn_i_(f)) of the instrumental noise (n_i_) affecting force cell measures was estimated by recording GRFs for a 20 s long session while belts were removed, sensorized surfaces were unloaded, and AP motors ran at 1.0 m/s. Results (Table 3) show that the standard deviation of n_i_ was below 0.14 N for all components. Moreover, as expected the total power of Gn_i_(f) was of low magnitude (below 0.05 W) demonstrating that the instrumental noise was negligible.

**Table 3:** Effect of the noise on GRF components

		**ML**		**AP**		**V**	
	**Speed [m/s]**	**mean (std) [N]**	**P [W]**	**mean (std) [N]**	**P [W]**	**mean (std) [N]**	**P [W]**
n_i_	1.00	−0.20 (0.09)	0.04	−0.23 (0.09)	0.05	0.01 (0.14)	0.02
	(without belts)						
n_m_	0.50	1.39 (0.27)	1.62	6.58 (0.78)	35.35	−0.12 (0.87)	0.79
	1.00	1.45 (0.53)	2.00	5.61 (0.93)	26.17	−0.22 (1.96)	3.84
	1.50	1.53 (0.83)	2.57	4.92 (1.71)	22.35	0.19 (1.82)-	3.28
	1.88	1.56 (1.52)	4.27	4.57 (1.34)	18.65	0.31 (2.16)	4.74

The table reports both mean and standard deviation (std) of GRF components, and the total power (P) of the power spectral density related to instrumental noise (n_i_) and noise due to moving parts (n_m_).

### Noise due to moving parts

The level of mechanical noise (n_m_) and related power spectral density, Gn_m_(f), generated by the AP motion of belts and other mechanical parts (i.e., rollers, motors) was estimated by recording GRFs for 20 s, with sensorized surface unloaded, at different speeds: 0.5, 1.0, 1.5 and 1.88 m/s. Results (Table 3) show that the amplitude of n_m_ was found to be dependent on the speed. In particular: concerning the vertical component of GRFs, the maximum standard deviation of n_m_ (2.16 N) was observed at the maximum speed (1.88 m/s); concerning ML and AP components of GRFs, the maximum standard deviation of n_m_ were respectively 1.52 N at 1.88 m/s, and 1.71 N at 1.50 m/s. The AP component was the one most affected by the movement of the belts and the mean of n_m_ increased with the speed.

The total power of Gn_m_(f) of the AP component was higher (less than 35 W) than those referring to ML and V ones (less than 5 W) at all speeds. Moreover the total power of Gn_m_(f) of the AP component decreased with the speed whereas those referring to ML and V components increased with the speed.

### Pilot tests

**Figure 9:**
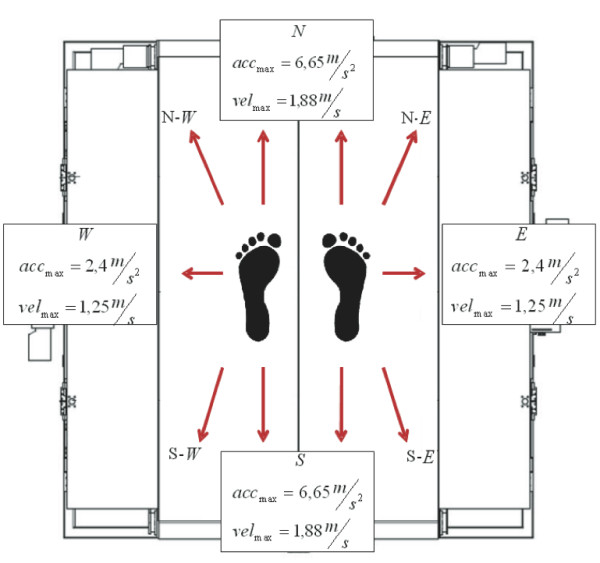
**Perturbations.** Kinematic features of the perturbations: North and South for both right and left feet; North-East, East and South-East for right foot; North-West, West and South-West for left foot. A perturbation consisted in: accelerating the belt up to the maximum speed, keeping the speed constant for a certain period, and stopping the belt. The N-W, N-E, S-W and S-E perturbations were obtained as a combination of the pure N, S, E and W ones.

In order to evaluate SENLY performance during real experimental sessions, a set of tests aimed at i) comparing the walking vertical GRFs obtained with data in literature, ii) verifying the capability of the asynchronous triggering to identify a specific event of the gait and iii) verifying the dynamic of the belts, was carried out. In particular, five healthy subjects (3 males and 2 females with an average age of 26 years) underwent ten different perturbations, as described in Figure 9, while walking on SENLY at normalized speed set in accordance with the Froude number (0.15). According to the asynchronous triggering, perturbations started when the ratio between the GRF vertical components measured under the perturbed foot and the contralateral component was 0.2 in order to destabilize during the early stance phase.

**Figure 10:**
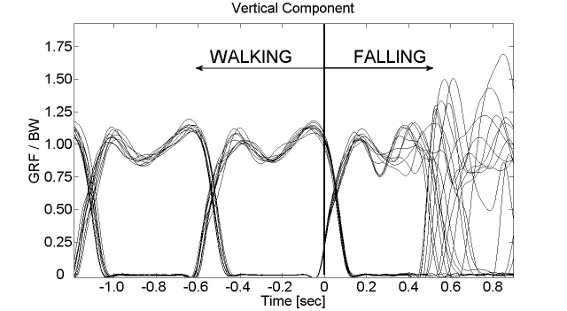
**Vertical component of the GRF during pilot tests.** GRF vertical components normalized to body weight while a subject underwent ten different perturbations occurring during the early stance phase. Walking speed was 1.11 m/s. In order to show the robustness of the algorithm triggering a specific event, all variables are aligned with respect to the trigger (Time = 0).

Measured GRFs were off-line low-pass filtered (cut off at 10 Hz) with a fourth-ordered, zero-phase lag, Butterworth filter. Results (Figure 10) showed that GRF trend measured during walking was comparable to data in literature [36].

The asynchronous triggering is thoroughly able to identify the required instant and allows users to provide perturbations with a standard procedure across many subjects (Figure 10).

**Figure 11:**
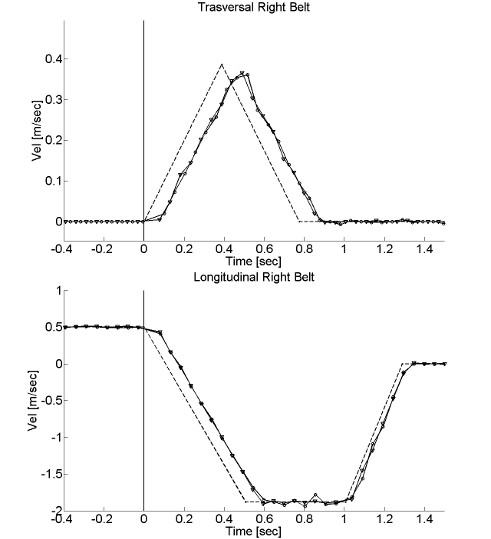
**Velocity profiles of the belts.** AP and ML velocity profiles of the right belt, during the unloaded case (round line), the loaded case (triangle marker), and the theoretical profiles (dash line).

To verify the accordance between kinematic specifications and the performance of SENLY, the speed profiles of both the AP and ML movements of the belts were analyzed both with and without a load (a 80 kg weighted subject carrying out experimental sessions). Results (Figure 11) show that, in all cases, both the AP and the ML velocity profiles of belts met required specifications with a delay of about 0.1 s due to the transitory dynamics.

## Conclusion

A new perturbing platform able to reproduce different slipping paradigms during various daily motor tasks (e.g., walking, upright standing) was designed and developed. One of its main features was the ability to adopt measured GRFs at run time in order to enable the asynchronous triggering of a specific event during a gait cycle. A customized procedure was achieved to improve precision and accuracy of GRF and COP measurements. Noticeably, the calibration procedure accounted for loading forces whose frequency bandwidths were comparable to those during locomotion, allowing users to suitably estimate the dynamical features of the load. Finally, the on-line analysis of GRFs and the related asynchronous triggering allows users to standardize experimental sessions, improving the efficacy of functional analysis.

## Competing interests

The authors declare that they have no competing interests.

## Authors’ contribution

All authors contributed to the design of SENLY, to draft and review the manuscript. LBL, VG, and EC contributed to the development of the platform. LBL and VG carried out the calibration and the testing of the platform. LBL, VG, and VM contributed to data analysis. LO and SM contributed in the supervision of the research study. All authors read and approved the final manuscript.
